# COMPARISON OF DIFFERENT CRITERIA IN THE PREVALENCE OF METABOLIC SYNDROME IN STUDENTS FROM PARANAVAÍ, PARANÁ

**DOI:** 10.1590/1984-0462/;2019;37;3;00007

**Published:** 2019-06-03

**Authors:** Flávio Ricardo Guilherme, Matheus Amarante do Nascimento, Carlos Alexandre Molena-Fernandes, Vânia Renata Guilherme, Stevan Ricardo dos Santos, Rui Gonçalves Marques Elias, Wilson Rinaldi

**Affiliations:** a Universidade Estadual do Paraná, Paranavaí, PR, Brazil.; b Faculdade de Tecnologia e Ciências do Norte do Paraná, Paranavaí, PR, Brazil.; c Centro Universitário Uningá, Maringá, PR, Brazil.; d Universidade Estadual do Norte do Paraná, Jacarezinho, PR, Brazil.; e Universidade Estadual de Maringá, Maringá, PR, Brazil.

**Keywords:** Metabolic syndrome, Obesity, Abdominal fat, Adolescent, Síndrome metabólica, Obesidade, Gordura abdominal, Adolescente

## Abstract

**Objective:**

To investigate the difference in the proportion of students with metabolic syndrome, diagnosed according to different criteria.

**Methods:**

The sample consisted of 241 students (136 boys and 105 girls) aged 10 to 14 years, from public and private schools in Paranavaí, Paraná. We used three distinct diagnostic criteria for metabolic syndrome, considering the presence of at least three of the following risk factors: increased waist circumference, hypertension, fasting hyperglycemia, low HDL-C, and elevated triglycerides.

**Results:**

The prevalence of metabolic syndrome found was 1.7% (confidence interval of 95% - 95%CI 0-3.3) for the IDF criterion; 3.3% (95%CI 1.0-5.6) for Cook; and 17.4% (95%CI 12.6-22.3) for Ferranti. Analyzing the criteria in pairs, the agreement between IDF and Cook was 97.5% (k=0.95); between IDF and Ferranti, 83.4% (k=0.67); and between Cook and Ferranti, 85.9% (k=0.72). Onlyone student (0.4%) was diagnosed with metabolic syndrome solely by the IDF criterion, while 34 (14.1%) were diagnosed exclusively by Ferranti. The comparison of the three criteria showed that Ferranti presented the highest proportion of metabolic syndrome (p<0.001), and Cook had a greater proportion than IDF (p<0.001).

**Conclusions:**

We found a significant difference in the proportion of metabolic syndrome in the three criteria. The choice of which criterion to use can compromise not only the percentage of metabolic syndrome prevalence but also interfere in strategies of intervention and prevention in children and adolescents with and without metabolic syndrome, respectively.

## RESUMO

**Objetivo:**

Investigar a diferença na proporção de escolares com síndrome metabólica diagnosticada segundo diferentes critérios.

**Métodos:**

Duzentos e quarenta e um escolares (136 meninos e 105meninas), com idade entre dez e 14 anos, das redes pública e privada de Paranavaí, Paraná. Foram utilizados três diferentes critérios para o diagnóstico da síndrome metabólica, considerando a presença de, ao menos, três dos seguintes fatores de risco: circunferência de cintura aumentada, hipertensão arterial, hiperglicemia em jejum, baixo nível de HDL-C e triglicerídeos elevado.

**Resultados:**

A prevalência de síndrome metabólica encontrada foi de 1,7% (intervalo de confiança de 95% - IC95% 0-3,3), para o critério de IDF; 3,3% (IC95% 1,0-5,6) em Cook; e 17,4% (IC95% 12,6-22,3) em Ferranti. Na verificação dos critérios em pares, a concordância entre IDF e Cook foi de 97,5% (k=0,95); entre IDF e Ferranti, 83,4% (k=0,67); e entre Cook e Ferranti, 85,9% (k=0,72). Em apenas um aluno (0,4%) a síndrome metabólica foi diagnosticada exclusivamente pelo critério de IDF, e em 34 alunos (14,1%), pelo critério de Ferranti. A comparação entre os três critérios mostrou que o de Ferranti apresentou maior proporção de síndrome metabólica que os demais (p<0,001), e o de Cook maior proporção em relação ao da IDF (p<0,001).

**Conclusões:**

Houve diferença significante na proporção de síndrome metabólica nos três critérios. A escolha do critério a ser utilizado pode comprometer não apenas o percentual de prevalência de síndrome metabólica, mas também atrapalhar as estratégias de prevenção e intervenção em crianças e adolescentes com e sem síndrome metabólica, respectivamente.

## INTRODUCTION

Metabolic syndrome (MS) is the association of at least three of the following risk factors: abdominal obesity, hypertension, hypertriglyceridemia, high levels of fasting blood glucose (FBG), and low levels of high-density lipoprotein (HDL-C).^1^Its prevalence increased in the past decade, and MS became a significant health issue worldwide, particularly in developing countries like Brazil.^2^Diagnosis is associated with the development of chronic diseases, especially cardiovascular ones and type 2 diabetes mellitus, regardless of age.^3^ ^,^ ^4^

Cut-off points for MS diagnosis in the adult population are well established,^5^ and several studies bring the prevalence and comparison with other populations, providing parameters of how MS is behaving in different parts of the world.^6^ ^,^ ^7^

Longitudinal studies have demonstrated that health issues begin in childhood and adolescence, justifying the investigation of MS and its risk factors in this period.^7^ ^,^ ^8^

However, in younger populations, the cut-off points have not yet been established, leading many studies to adapt MS definitions for adults to use in children and adolescents. Therefore,the identification of risk factors and, consequently, the MS prevalence vary considerably among the different criteria.^4^

The main reasons for the heterogeneity of criteria are the changes in growth and development during childhood and adolescence, resulting in cut-off points with no set values, particularly regarding blood pressure, lipids, and waist circumference.^9^ ^,^ ^10^ ^,^ ^11^The divergence is such that some studies have shown MS prevalence between 20 and 300% in the same sample.^12^ ^,^ ^13^

Thus, the objective of this study was to investigate the difference in the proportion of students with MS, diagnosed according to different criteria.

## METHOD

This cross-sectional study was conducted in July and August 2013. The sample design to investigate MS specifically was defined based on the total number of students (n=4,540); unknown prevalence; confidence level of 95%; and sampling error of 4%, leading to a minimum number of participants estimated in 206. Students were chosen by systematic random sampling, in three stages:

- Drawing of a school in each region of the city.
- Drawing of classes in each school.
- Invitation to all students of the selected classes and explanations about the study.

Consequently, 566 students aged 10 to 14 years, from 6th to 9th grade of public (4) and private (2) schools were selected and presented the informed consent form signed by parents or legal guardians. Among them, 325 individuals were excluded as they did not undergo all the necessary evaluations for MS diagnosis. The final sample comprised 241 children and adolescents, 136 boys and 105 girls. The margin of sampling error calculated *a posteriori* was 3.6 to 3.7%, below the value established *a priori* (4%).

Waist circumference was measured immediately above the iliac crests with a flexible and inextensible tape (Gulick, Brazil), with a resolution of 0.1 cm.^14^

Blood pressure measurement complied with the techniques recommended by the Brazilian Society of Cardiology,^15^ using a mercury sphygmomanometer (WanMed, Brazil). Three measurements were taken with a minimum interval of two minutes, considering valid the mean value between the last two.

To classify waist circumference and blood pressure variables, whose abnormalities are diagnosed according to their distribution in percentiles, we used references by Fernandez et al.^16^and The Fourth Report on the Diagnosis, Evaluation, and Treatment of High Blood Pressure in Children and Adolescents.^17^

Samples of 10 mL of venous blood from the antecubital vein were collected for biochemical analyses, after a fasting period of at least 10-12 hours, between 8:00 and 9:30a.m., in a clinical analysis laboratory of the city, and analyzed on the same day. Tests included fasting blood glucose and lipid profile, which consisted of serum levels of total cholesterol, HDL-C, low-density lipoprotein cholesterol (LDL-C), andtriglycerides.

We used three criteria to identify MS, two based on the National Cholesterol Education Program, modified for children and adolescents by Cook et al.^18^ and Ferranti et al.;^19^ and the third on the consensus proposed by the International Diabetes Federation (IDF) (Table 1).^1^

**Table 1:** Variables and cut-off points according to different classifications of metabolic syndrome.

Criteria	WC	BP	FBG (mg/dL)	HDL-C (mg/dL)	TG (mg/dL)
Cook et al.^18^	≥P90	≥P90	≥110	≤40	≥110
Ferranti et al.^19^	≥P75	≥P90	≥110	≤50	≥100
IDF (2007) (Zimmetet al.^1^)	≥P90	SBP≥130 mmHg orDBP≥85 mmHg	≥100	≤40	≥150

WC: waist circumference; BP: blood pressure; FBG: fasting blood glucose; HDL-C: cholesterol within high-density lipoprotein; TG: triglycerides; IDF: International Diabetes Federation; P: percentile; SBP: systolic blood pressure; DBP: diastolic blood pressure.

The statistical analysis tested the normality of data using the Kolmogorov-Smirnov test, and the existence of outliersthrough box plots.We included outliers in the analyses because they corresponded to data of subjects with anthropometric and metabolic changes of interest for the study. For continuous variables, we used descriptive analysis - percentiles (P25, P50, P75, P90), mean (confidence interval of 95% - 95%CI), and standard deviation (SD). Proportions of categorical variables were compared by the chi-square test and Fisher’s exact test. We calculated the Kappa index to verify the agreement between results obtained from the different diagnostic criteria. Due to the asymmetry of data distribution in the contingency table, which compromises the interpretation and calculation of Kappa, we used the prevalence and bias adjusted Kappa (PABAK). The significance level adopted for all tests was p<0.05.

## RESULTS

Table 2 presents the general characteristics of the sample, as well as the confidence interval of the means of variables. Thesample consisted of 241 children and adolescents with a mean age of 12.3±1.2 years, 136 (56.4%) females, 134 (55.6%) aged 10-12 years, and 107 (44.4%) aged 13-14 years.

**Table 2:** Sample description according to anthropometric characteristics, blood pressure, and metabolic variables of students from Paranavaí, Paraná, 2013.

	P25	P50	P75	P90	Mean±SD (95%CI)
Age (years)	11	12	13	14	12.3±1.2 (12.2-12.5)
Weight (kg)	42.2	49.4	57.2	65	50.1±12.0 (48.6-51.6)
Height (cm)	1.51	1.58	1.64	1.7	1.58±0.1 (1.57-1.59)
BMI (kg/m^2^)	17.4	19.5	21.8	25.7	20±3.6 (19.5-20.4)
WC (cm)	65	71	78.5	86.8	72.3±10.3 (71.0-73.7)
SBP (mmHg)	100	111	122.5	131	110.9±17.1 (108.7-103.1)
DBP (mmHg)	58	63	72.5	81	65.2±12.2 (63.7-66.8)
Blood glucose (mg/dL)	65.4	76.9	93.6	100.9	78.9±16.8 (76.8-81.0)
Cholesterol (mg/dL)	177.3	201.9	236.4	271.1	205.7±44.7 (200.0-211.3)
LDL-C (mg/dL)	47	61.7	79.3	109.5	58±38.0 (53.2-62.8)
HDL-C (mg/dL)	44.5	49.9	54.2	58.1	49.4±6.8 (48.5-50.2)
TG (mg/dL)	60.6	75	99.3	138.4	87.8±44.7 (82.1-93.5)

P: percentile; SD: standard deviation; 95%CI: confidence interval of 95%; BMI: body mass index; WC: waist circumference; SBP: systolic blood pressure; DBP: diastolic blood pressure; LDL-C: cholesterol within low-density lipoprotein; HDL-C: cholesterol within high-density lipoprotein; TG: triglycerides.

Table 3 shows the proportion of positive MS diagnosis, according to the criteria used. The prevalence of MS found was 1.7% (95%CI 0-3.3%) for the IDF criterion, 3.3% (95%CI 1.0-5.6%) for Cook, and 17.4% (95%CI 12.6-22.3%) for Ferranti. The agreement analysis revealed that three students (1.3%) had the same MS diagnosis in the three definitions. Analyzing the criteria in pairs, the agreement between the IDF and Cook was 97.5% (adjusted k=0.95). Between IDF and Ferranti, the agreement was 83.4% (adjusted k=0.67), and between Cook and Ferranti, 85.9% (adjusted k=0.72). Only one student (0.4%) was diagnosed with MS solely by the IDF criterion, while 34 (14.1%) were diagnosed exclusively by Ferranti. The comparison among the three criteria by the chi-square test and Fisher’s exact test showed that Ferranti presented the highest proportion of MS (p≤0.001), and Cook had a greater proportion than IDF (p≤0.001).

**Table 3:** Proportion of students according to metabolic syndrome and number of risk factors in the three criteria.

	Cook et al.^18^	Ferranti et al.^19^	IDF (2007) (Zimmet et al.^1^)
MS prevalence (95%CI) (n=241)	3.3% (1-5.6)^*#^	17.4% (12.6-22.3)^*†^	1.7%; (0-3.3)^#†^
Age			
10-12 years (n=134)	1.5% (0-3.6)	17.9% (11.3-24.5)	1.5% (0-3.6)
13-14 years (n=107)	5.6% (1.2-10)	16.8% (9.6-24)	1.9% (0-4.5)
Risk factors			
0	44.4% (38.6-50.2)	18.7% (13.7-24.1)	55.2% (49-61.4)
1	36.5% (30.7-42.3)	39% (33.2-45.6)	32.4% (26.2-39)
2	15.8% (11.6-20.3)	24.9% (19.9-30.3)	10.8% (7.1-14.9)

IDF: International Diabetes Federation; 95%CI: confidence interval of 95%; MS: metabolic syndrome; ^*^k=0.28; adjusted k=0.72; ^#^k=0.49; adjusted k= 0.95; ^†^k=0.10; adjusted k=0.67.

Regarding the analysis of the proportion of components in each criterion, Ferranti was the most distinct among the three, having a lower proportion in students with no risk factor, as well as higher proportion among students with three or more risk factors (p≤0.001). The comparison between Cook and IDF showed a significant difference (p≤0.001); however, percentage values for each number of components identified presented less variation in their respective criteria.

**Figure 1:**
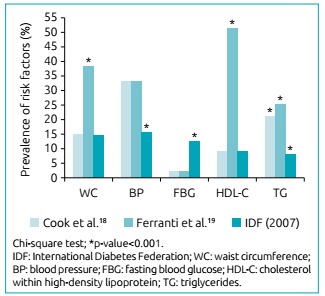
Prevalence of abnormal risk factors for diagnosis of metabolic syndrome.

The separate analysis of MS components demonstrated that Ferranti’s criterion had proportions of increased waist circumference and inadequate HDL-C values significantly higher than the other two criteria (p≤0.001). The IDF criterion had the lowest proportion in the blood pressure (p≤0.001) and triglycerides (p<0.001) components. With respect to blood glucose, the IDF criterion presented the highest prevalence (p≤0.001) (Figure 1).

## DISCUSSION

Due to divergences in the literature regarding the definition of MS in children and adolescents, studies involving this population have adapted criteria and cut-off points for age and gender to try to diagnose these individuals.^10^ ^,^ ^12^ ^,^ ^13^ ^,^ ^20^ ^,^ ^21^

Attempting to verify differences in proportions of MS among students based on three different criteria, the present study revealed that the criterion proposed by IDF had the lowest prevalence (1.7%), followed by Cook (3.4%), and Ferranti (17.4%). Otherstudies also showed that Ferranti’s criterion presented a higher MS prevalence compared to other parameters, corroborating our finding.^10^ ^,^ ^22^ A possible explanation is the fact that its cut-off points for waist circumference and triglycerides are less strict.

Among risk factor components, fasting hyperglycemia had the lowest prevalence. The largest proportion of this variable was found in the IDF criterion (12.4%); in the other two, only 1.7% of the sample was diagnosed. A study with obese adolescents using the same criteria of this investigation presented similar results, revealing a higher percentage of elevated blood glucose in the IDF criterion (7.4%) and lower (1.7%) in the other ones.^10^

Blood glucose was also the least prevalent variable among MS risk factors in several studies that used other criteria to diagnose MS.^23^ ^,^ ^24^ This fact puts in question the use of blood glucose as a risk factor component to detect MS. Some researchers have suggested adopting the Homeostatic Model Assessment for Insulin Resistance (HOMA-IR) instead of fasting blood glucose,^25^ as this test verifies insulin resistance, which precedes hyperglycemia, and is more indicated for this population.

The component with the highest prevalence was different in the three criteria: in Ferranti, it was low HDL-C, and in Cook and IDF, hypertension; contrary to the findings of other studies,^10^ ^,^ ^21^ ^,^ ^24^ ^,^ ^26^ ^,^ ^27^ which identified waist circumference as the most prevalent component, regardless of the criteria used.

One of the reasons for the proportion of waist circumference being higher in these studies might be the fact that only overweight and/or obese children and adolescents - identified with body mass index (BMI) - were analyzed. It is clear in the literature that BMI has a very strong correlation with waist circumference in this age group,^28^ causing a large part of the sample studied to be also classified with central obesity, thus justifying why waist circumference was the component with the highest prevalence.

Despite the strong association of this anthropometric index with cardiovascular diseases and MS,^29^we emphasize that MS is not diagnosed only by the presence of abdominal obesity. In this regard, studies with samples in different nutritional states that compare diagnostic criteria for MS are necessary to demonstrate this issue better.

Regarding blood pressure, the IDF criterion had the lowest prevalence of hypertensive individuals (15.4%), while the remaining criteria had 32.8% prevalence, a similar result to the one found in the study with obese adolescents in the same age group (10-14 years) from Porto Alegre, Rio Grande do Sul.^10^The IDF criterion uses higher cut-off points and does not classify adolescents according to age, gender, and height, which could explain the result found.

Although the objective of this study is the presence of MS based on different diagnostic criteria, we underline the proportion of students who showed one and two risk factors. Forinstance, 63.9% of students presented one or two risk factors in Ferranti, 52.3% in Cook, and 43.2% in IDF, a difference considered significant (p≤0.001). Similarly, studies have found a high prevalence of risk factors in children and adolescents based on different criteria.^10^ ^,^ ^20^ ^,^ ^30^ Considering that our sample consisted of students aged 10 to 14 years and that some changes might not yet have manifested, thehigh prevalence of these factors could result in their persistence until adulthood - fact known as tracking of MS^4^ - and/or the emergence of new risk factors over the years, which could lead to a future MS diagnosis.

The present study had some limitations, such as not evaluating the technical error of measurements and the coefficient of variation between evaluators, particularly in waist circumference and blood pressure measurements. Also, it did not stratify the sample according to the maturity level, a variable that can influence MS risk factors. In contrast, this study provides important practical applications for health professionals who work with prevention and control of risk factors and MS in adolescents, as based on the findings of this investigation, they will know that, depending on the criterion adopted to diagnose MS, the confirmation of risk factors, and, consequently, the MS diagnosis might be different.

Considering that we found significant difference in MS diagnosis among the three criteria used, as well as in the proportion of components and number of risk factors, the choice of which criterion to use can compromise not only the percentage of MS prevalence but also interfere in strategies of intervention and prevention in children and adolescents with and without MS, respectively. Thus, establishing specific cut-off points to diagnose MS in children and adolescents is necessary, given the differences found in this study and the literature regarding the interpretation and comparison of results in different samples.
